# 

**Published:** 1856-10

**Authors:** 


### THE JOURNAL OF PUBLIC HEALTH.

### CONTENTS OF No. VII.

Air and Ventilation........................................ 193

Ozone........................................ 218

**EPITOME OF SANITARY LITERATURE:**

Smoke Nuisance........................................ 221

Preventive Medicine........................................ *ib.*

Lunacy Laws and Asylums........................................ *ib.*

Animal Diet........................................ 222

Turnbull on Diseases of the Stomach........................................ *ib.*

Sanitary Reports........................................ *ib.*

**ORIGINAL COMMUNICATIONS:**

The Patriarchs of Pinner. By John Webster, M.D., F.R.S. ............ 223

The Territorial Distribution of the Population for Purposes of Sanitary Inquiry and Social Economy. By Henry W. Rumsey, Esq........... 225

Cholera and the Water Supply in the South Districts of London in 1854. By John Snow, M.D........................................ 239

The Hygienic Treatment of Pulmonary Consumption. By B. W. Richardson, M.D........................................ 258

**SANITARY AND SOCIAL SCIENCE:**

*Reports on Cities, Towns, and Districts.*—Dudley........................................ 287

Miscellanea.—A New Gas Stove........................................ 293

**PROGRESS OF EPIDEMICS in Thirty-Six Towns and Districts**............ 294

**SANITARY LEGISLATION:**

Sale of Bad Meat in London.—The Census of Ireland.—Report on the Adulteration of Food........................................ 311

**TRANSACTIONS of the EPIDEMIOLOGICAL SOCIETY of London:**

Annual Report of the Council........................................ 73

On Concurrent Scarlet Fever and Measles. By W. B. Kesteven, Esq. .... 80

On the Occurrence of Fever at Cowbridge in 1853. By William Camps, M.D. 83

Quarterly Report of Proceedings........................................ 87

